# A framework for multi-component analysis of diffusion MRI data over the neonatal period

**DOI:** 10.1016/j.neuroimage.2018.10.060

**Published:** 2019-02-01

**Authors:** Maximilian Pietsch, Daan Christiaens, Jana Hutter, Lucilio Cordero-Grande, Anthony N. Price, Emer Hughes, A. David Edwards, Joseph V. Hajnal, Serena J. Counsell, J-Donald Tournier

**Affiliations:** a Centre for the Developing Brain, School of Bioengineering and Imaging Sciences, Kings College London, Kings Health Partners, St. Thomas Hospital, London, SE1 7EH, UK; b Department of Biomedical Engineering, School of Bioengineering and Imaging Sciences, Kings College London, Kings Health Partners, St. Thomas Hospital, London, SE1 7EH, UK

**Keywords:** Diffusion MRI, Brain development, White matter, Atlas

## Abstract

We describe a framework for creating a time-resolved group average template of the developing brain using advanced multi-shell high angular resolution diffusion imaging data, for use in group voxel or fixel-wise analysis, atlas-building, and related applications. This relies on the recently proposed multi-shell multi-tissue constrained spherical deconvolution (MSMT-CSD) technique. We decompose the signal into one isotropic component and two anisotropic components, with response functions estimated from cerebrospinal fluid and white matter in the youngest and oldest participant groups, respectively. We build an orientationally-resolved template of those tissue components from data acquired from 113 babies between 33 and 44 weeks postmenstrual age, imaged as part of the Developing Human Connectome Project. These data were split into weekly groups, and registered to the corresponding group average templates using a previously-proposed non-linear diffeomorphic registration framework, designed to align orientation density functions (ODF). This framework was extended to allow the use of the multiple contrasts provided by the multi-tissue decomposition, and shown to provide superior alignment. Finally, the weekly templates were registered to the same common template to facilitate investigations into the evolution of the different components as a function of age. The resulting multi-tissue atlas provides insights into brain development and accompanying changes in microstructure, and forms the basis for future longitudinal investigations into healthy and pathological white matter maturation.

## 1 Introduction

Preterm birth and associated complications are the leading cause of child mortality worldwide, accounting for 965,000 neonatal deaths annually (Liu et al., 2015). The prevalence of preterm birth ranges from 5% to 18%, on average 11.1% with an increasing incidence (Blencowe et al., 2012). Preterm birth raises the risk of abnormal brain development, resulting in impaired motor and cognitive function, cerebral palsy, learning disability and life-long chronic disease impacting families and societies (Mwaniki et al., 2012; Volpe, 2001, 2009; Miller et al., 2005; Ferriero, 2004; Shankaran et al., 2015).

MRI has become the modality of choice for studying the developing brain in a non-invasive way. During the neonatal period, the human brain increases in size rapidly (Brody et al., 1987) and cerebral tissue undergoes marked changes in cellular composition, density and water content (Dobbing and Sands, 1973). Neuroimaging can play a crucial role in the characterisation and diagnosis of abnormal brain maturation, guide counselling and inform on possible prevention and the development of treatments (Molnár and Rutherford, 2013; Kwon et al., 2014; Counsell et al., 2014). This requires the development of specific imaging markers that capture developmental processes such as cell proliferation, migration, and maturation.

Acquisition, processing and analysis of neonatal diffusion data is challenging due to this modality's sensitivity to motion and the comparatively low signal to noise ratio, especially in neonates with relatively high overall free water content. Recent developments in diffusion MRI acquisition strategies now allow the routine collection of large amounts of data (Larkman et al., 2001). This makes it possible to acquire eloquent multi-shell HARDI data in neonates within acceptable scan times, providing microstructural information about the developing brain not available using other imaging modalities.

### 1.1 Prior work on neonatal microstructural analysis

Prior work in neonatal group-analysis of diffusion data aims to establish and improve inter-subject correspondence, increase sensitivity and robustness to large shape and intensity changes during development, increase specificity to tissue classes and white matter (WM) fibre tracts, and to build models with interpretable parameters with respect to biological tissue properties. A comprehensive review of diffusion models and analysis methods is beyond the scope of this manuscript, see (Novikov et al., 2018) for a review on microstructure modelling in diffusion MRI (Dell’Acqua and Tournier, 2018), for a discussion of spherical deconvolution techniques, and (Pecheva et al., 2018) for a summary of analysis techniques applied to neonatal data.

Neonatal diffusion MRI studies often involve: the statistical analysis of aggregate values from manually drawn regions of interest (Hüppi et al., 1998; Gao et al., 2009), atlas-based definition of anatomically or functionally coherent regions, voxel-based morphometry (Ashburner and Friston, 2000), tractography-guided definition of tracts of interest (Reijmer et al., 2012; Taylor et al., 2015; Shi et al., 2014), tract-based spatial statistics (Smith et al., 2006) and tract-specific analysis (Yushkevich et al., 2009; Pecheva et al., 2017). These types of analysis typically rely on the creation or existence of group average templates to establish spatial correspondence between subjects and for statistical analysis.

Diffusion tensor imaging has been used widely to study white matter and brain development (Hüppi and Dubois, 2006; Peters et al., 2012; Yoshida et al., 2013; Dubois et al., 2014; Ouyang et al., 2018; Rose et al., 2014; Pannek et al., 2013; Huang et al., 2013). However, this model comes with a number of limitations (Jones et al., 2013), in particular its limited capacity to capture microstructural information, its inability to resolve multiple fibre populations, and its sensitivity to partial volume effects with cerebrospinal fluid (CSF).

Recently, the availability of multi-shell HARDI data has allowed the use of higher order diffusion models in the developing brain (Kunz et al., 2014; Genc et al., 2017; Dean et al., 2017; Batalle et al., 2017; Pannek et al., 2018). There are a large number of higher order diffusion microstructure models that attempt to model biological tissue properties beyond the tensor model (see Novikov et al., 2018; Ferizi et al., 2017; Jelescu and Budde, 2017)). However, such models are based on (bio-)physical models of mature white matter, which are unlikely to be applicable to the neonatal case. Currently, there is no model that is specific to neonatal brain maturation.

In (Dean et al., 2017), the NODDI framework (Zhang et al., 2012) is used to extract time-resolved regional patterns in the developing brain, using a group-average fractional anisotropy (FA) template for spatial alignment. Pannek et al. use constrained spherical deconvolution (Tournier et al., 2007) with a *response function* derived from WM and orientation distribution function (ODF)-based groupwise registration (Raffelt et al., 2011) to investigate group differences in micro- and macroscopic tissue differences between term-born infants and infants born preterm at term equivalent age (Pannek et al., 2018). Compared to diffusion tensor imaging, fibre ODF-based analysis using the constrained spherical deconvolution (CSD) technique (Tournier et al., 2007) allows delineating more complex WM bundles in neonates (Pecheva et al., 2018). Nevertheless, neonatal CSD-based analysis has been limited to a single WM-based contrast, not taking the temporal evolution of the WM signal into account.

The fixel-based analysis framework (Raffelt et al., 2017a) is based on fibre ODFs and addresses the limitations of scalar, voxel-based analysis. It allows the statistical analysis of WM tracts in the presence of multiple fibre populations per voxel. Statistical significance can be boosted by aggregating information from connected voxels via tractography and cluster free threshold enhancement (Smith and Nichols, 2009). In neonatal and infant data, this technique allows the cross-sectional (Pannek et al., 2018) and longitudinal (Genc et al., 2018) analysis of fibre density and morphology.

### 1.2 Existing neonatal atlases

In neuroimaging, most group-analysis methods require spatial correspondence between subjects, which is frequently achieved via registration to study-specific group templates, or external atlases (Hess et al., 2018; Oishi et al., 2018). In recent years, a number of publications have focused on creating mappings of microstructural and anatomical properties of the developing brain using T_1_ and T_2_-weighted imaging (Shi et al., 2014; Dittrich et al., 2014; Schuh et al., 2014, 2018; Habas et al., 2009; Kuklisova-Murgasova et al., 2011; Serag et al., 2012a, 2012b). Neonatal atlases of subjects aged 38 weeks or older using T_1_ or T_2_-weighted images are reported in (Kazemi et al., 2007; Noorizadeh et al., 2013; Ghadimi et al., 2017; Alexander et al., 2017; Shi et al., 2011, 2014; Zhang et al., 2016a, 2016b; Li et al., 2015; Oishi et al., 2011; Gousias et al., 2012), and have also included co-registered CT images (Ghadimi et al., 2017), or FA and mean diffusivity maps (Oishi et al., 2011). Anatomical atlases including very preterm subjects have been published in (Kuklisova-Murgasova et al., 2011; Serag et al., 2012b; Schuh et al., 2014, 2018; Makropoulos et al., 2016). See (Schuh et al., 2018; Benkarim et al., 2017) for recent reviews of neonatal atlases with a focus on structural imaging and temporal modelling and (Oishi et al., 2018) for a review covering brain parcellation atlases.

Relaxation-based neonatal atlases provide high-resolution anatomical maps with contrast sensitive to the changes observed during development. Diffusion-weighted imaging offers additional information about microstructural tissue properties relevant for characterising neonatal development (Yoshida et al., 2013). Furthermore, diffusion-based atlases can provide orientationally resolved information about the microstructure of individual fibre populations not discernible with other modalities and has the potential to be used to directly and simultaneously assess the organisation and microscopic properties of individual fascicles in the developing brain.

In neuroimaging, using standardised brain atlases as a reference space for analysis facilitates cross-study comparison of results. However a study-specific template is likely to increase the analysis’ sensitivity and specificity (Huang et al., 2010; Van Hecke et al., 2011; Zhang and Arfanakis, 2013). For the analysis of diffusion properties, it is in general desirable to use the diffusion images or parameter maps derived from them for image registration instead of anatomical reference images as this directly optimises alignment of the contrast of interest.

Neonatal diffusion MRI (dMRI) atlases have typically been created using the diffusion tensor model (Oishi et al., 2011; Blesa et al., 2016; Otsuka et al., 2017; Yu et al., 2016; Akazawa et al., 2016). HARDI and higher order diffusion model-based atlases can alleviate some of the limitations of diffusion tensor-based atlases, yet comparatively few atlases of the neonatal brain have been created using HARDI data. Existing atlases consist of the averaged raw diffusion spectrum imaging (Yeh and Tseng, 2011) or HARDI data (Kim et al., 2017; Saghafi et al., 2016), or use reconstructed fibre ODFs (Shen et al., 2017), or tractograms (Zhang et al., 2018) to represent brain tissue and white matter. In comparison to adult HARDI data, neonatal data is more challenging to decompose into biologically meaningful and interpretable components such as WM and grey matter (GM). To date, no neonatal diffusion-based atlas has been built using multiple tissue contrasts or features specific to tissue maturation.

### 1.3 Registration and image fusion techniques

A group-average template allows the spatial alignment of information across the cohort and facilitates subsequent group-level and subject-level analysis and processing (Evans et al., 2012). In general, no registration and template building technique is considered optimal for generating a representative and unbiased atlas of a population, irrespective of the application and target domain (Evans et al., 2012). Within the listed neonatal structural atlases, a variety of registration approaches have been proposed, ranging from pairwise affine (Kuklisova-Murgasova et al., 2011), pairwise non-rigid (Serag et al., 2012a, 2012b; Schuh et al., 2014), to groupwise registrations (Shi et al., 2011; Alexander et al., 2017; Zhang et al., 2016a; Gholipour et al., 2017; Dittrich et al., 2014; Schuh et al., 2018). Pairwise registration approaches require accurate mapping between each subject and all other subjects, which is computationally demanding. Alternatively, each subject can be registered to an initial age-matched group-average template, which is subsequently refined iteratively.

Temporal patterns in foetal and neonatal brain MRI have been modelled based on image intensity, on deformations, or combinations thereof. In (Habas et al., 2009), a temporal probabilistic model of tissue classes has been used to express spatio-temporal patterns in foetal structural MRI. Temporal shape consistency can be modelled via kernel regression of rigid and non-rigid transformations (Davis et al., 2010; Kuklisova-Murgasova et al., 2011; Dittrich et al., 2014). This has been extended by approaches that use adaptive kernel sizes, which take variable sample densities in the temporal domain into account (Serag et al., 2012b; Schuh et al., 2014, 2018; Makropoulos et al., 2016; Zhang et al., 2016a; Gholipour et al., 2017).

Following registration, a template representative of the cohort can be created by (possibly weighted) averaging of the transformed images or via sparse patch-based image fusion techniques. Sparse template creation techniques can reduce blurring due to anatomical variability or misalignment but possibly introduce inconsistencies between adjacent or distant patches. Shi et al. use group sparsity constrained patch-based representations to recover anatomical detail beyond that achievable with image registration (Shi et al., 2014). An extension to this technique is spatio-temporal matching, which can be used to extract temporally consistent and spatially local patterns from the cohort (Zhang et al., 2016a). In (Saghafi et al., 2016), spatio-temporal regularisation is applied in the angular domain of the dMRI data. Kim et al. build a spatio-temporal graph of image patches and create the template from a sparse subset of this graph (Kim et al., 2017).

### 1.4 Overview of study

In this work, we describe a framework for building an unbiased, time- and orientation-resolved group average template of WM maturation based on advanced diffusion MRI methods, in a cohort of neonates scanned over a range of ages, during which large changes in brain volume, shape and contrast occur. We apply this framework on high-quality multi-shell HARDI data acquired as part of The Developing Human Connectome Project (dHCP)^1^ to build an atlas of 113 babies scanned just after birth with postmenstrual age (PMA) at scan ranging from 32.4 to 44.6 weeks.

The analysis of these microstructural properties in the developing brain requires two main components: a consistent model for the HARDI signal suitable for the neonatal period; and a means of aligning these data onto an unbiased common space. The model used in this work relies on the multi-shell multi-tissue constrained spherical deconvolution (MSMT-CSD) framework (Jeurissen et al., 2014) and requires the determination of appropriate *response functions* to describe the signal ‘signature’ for each different tissue component. The image registration is driven based on two such tissue components (Pietsch et al., 2017a, 2017b), to align subjects within 12 multi-tissue cross-sectional weekly templates, and then jointly to a single multi-tissue template which is split after alignment into weekly time steps. The atlas itself was created using a decomposition of the signal into three components: one isotropic, derived from CSF, and two anisotropic components. The latter are derived from the WM signal in the 9 subjects part of the youngest weekly group and 11 subjects part of the oldest weekly group, respectively. The resulting atlas provides a basis for detailed spatio-temporal investigations into healthy and abnormal brain maturation at the single fibre level.

Our approach contributes to different aspects of the neonatal analysis pipeline. We use a rich, multi-component, orientation-resolved representation that captures the majority of the HARDI signal and can be used to infer fibre-specific information. To characterise the WM maturation in a fibre-resolved manner, we utilise a data-driven representation instead of a biophysically motivated diffusion model.

To demonstrate this multi-component decomposition and multi-channel registration, we create a population-specific atlas of the developing brain using state-of-the-art HARDI data sampled over four diffusion weighting shells from the dHCP. To our knowledge, this is the highest quality in-vivo neonatal HARDI atlas to date, offering an unprecedented time-span, angular and spatial resolution. Our framework is compatible with any commonly used analysis framework, in particular fixel-based analysis. Hence, our proposed framework allows fibre-resolved investigations of normal and abnormal brain development. The atlas and related data will be available from http://brain-development.org/brain-atlases/.

## 2 Materials and methods

### 2.1 Cohort

The cohort used for this atlas consists of 113 babies scanned as part of the dHCP. From all subjects available, subjects with known clinical abnormalities (Hughes et al., 2017a) and lesions (using apparent diffusion coefficient (ADC), WM and CSF decomposition images) were excluded. If a subject was scanned multiple times, only the first scan was considered. The weekly cohorts have an average gestational age at scan of 32.9, 34.0, 35.2, 35.7, 37.1, 38.1, 39.1, 40.1, 40.9, 42.0, 42.8 and 44.1 weeks and consist of 11 subjects, except for the two youngest cohorts and the template at 35.7 weeks, which consist of 9, 9 and 10 samples, respectively.

We chose to include subjects from the healthy appearing dHCP cohort in this work, and did so in a way that strives to minimise any bias of the weekly templates on the basis of gender, age since birth and anatomy. To ensure comparable anatomical variability across age, we selected 9 to 11 subjects per weekly template. If more datasets were available for a template, we ranked them using the following criteria and chose only the 11 best samples.

**Figure A.13:**
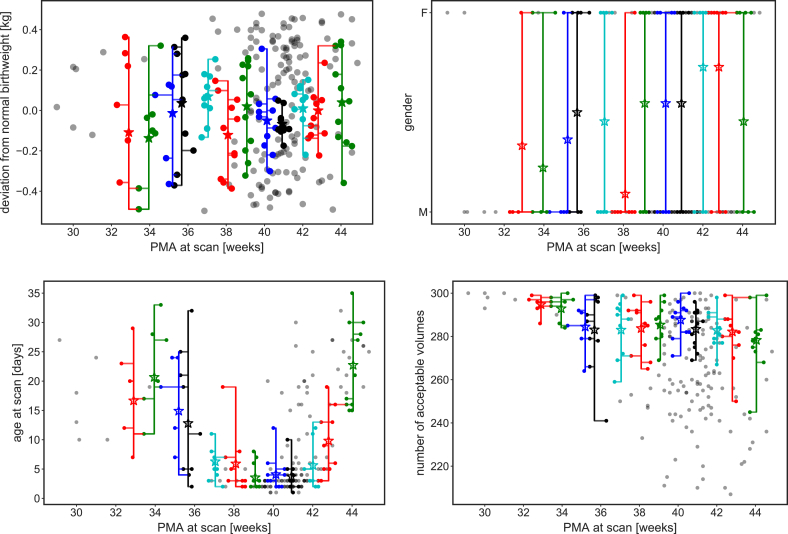
Demographics of the cohort. Lines link subjects that were grouped to build the weekly templates. Asterisks indicate the average for each template. Subjects that are part of the dHCP but were not used for this work are shown as grey dots.

The images were grouped so that the number of motion artefact free volumes per subject is maximised while minimising both the deviation from normal (age and gender-matched) birth weight and the age since birth. To better balance age range and gender-bias, subjects were assigned to up to two time points. See figures A.13 for plots of cohort age, weight and quality measures.

### 2.2 Data

The multi-shell high angular resolution diffusion single-shot spin-echo echo-planar images were acquired on a Philips 3T Achieva scanner using a dedicated neonatal head coil (Hughes et al., 2017b) with a maximum gradient amplitude of 70 mT/m. The 300 volumes per image were sampled with four phase-encode directions on four shells with b-values of 0 (n = 20), 400 (n = 64), 1000 (n = 88) and 2600 (n = 128) with TE = 90, TR = 3800 ms (Tournier et al., 2015; Hutter et al., 2018) and reconstructed to a resolution of 1.5 mm. The reconstruction method follows the extended SENSE technique proposed in (Zhu et al., 2016). Sensitivities were estimated from non-accelerated reference acquisitions with matched readouts as in (Hennel et al., 2016) to promote equivalent distortions in the coil maps as in the data.

### 2.3 Preprocessing

The preprocessing of the data consists of: (i) removal of motion-corrupted volumes using a deep neural network classifier (Kelly et al., 2017); (ii) Marchenko-Pastur-PCA-based denoising (Veraart et al., 2016) (MRtrix3); (iii) susceptibility and eddy-current distortion correction and inter-volume motion correction with outlier replacement using *topup* (Andersson et al., 2003) (FSL) and *eddy* (Andersson and Sotiropoulos, 2016) (FSL); and (iv) bias field correction based on the b = 0 shell using *N4* (Tustison et al., 2010) (ANTs).

Brain masks were generated using a combination of *bet* (Smith, 2002) (FSL) and a custom-built threshold-based segmentation.

### 2.4 Tissue decomposition

The approach of decomposing the diffusion signal used in this study relies on the MSMT-CSD technique (Jeurissen et al., 2014), which separates the diffusion signal into distinct, orientationally-resolved tissue types. MSMT-CSD requires multiple, component-specific *response functions* to be defined, each of which characterises the signal for the corresponding tissue component within each b-value shell, along with its angular dependence. Response functions can be either estimated from the data (Jeurissen et al., 2014; Christiaens et al., 2016; Dhollander et al., 2016; Dell’Acqua and Tournier, 2018) or modelled with a given functional form (White et al., 2013; Yap et al., 2016; Anderson, 2005; Descoteaux et al., 2009; Jespersen et al., 2007). These responses are then used to deconvolve the signal into multiple tissue-specific orientation distribution functions.

**Fig. 1:**
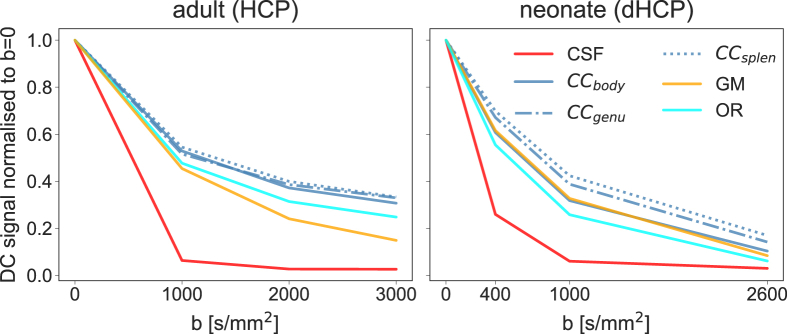
Mean (DC) signal sampled in CSF, WM and GM in adults and neonates at term-equivalent age (40 weeks PMA). In adults, GM and WM are separable by their average signal decay curves. This is not the case for neonates at term-equivalent age where the GM curve lies within the spectrum of WM curves and is very similar to the average signal decay in the body of the CC. Adult data was taken from the human connectome project (HCP). CC_splen_, CC_body_ and CC_genu_ correspond to the splenium, midbody and genu of the corpus callosum respectively; OR corresponds to the optic radiations.

In adults, the main feature that allows this separation is the fact that different tissue types have sufficiently distinct b-value dependencies, giving a clear separation of the signal into WM, GM and CSF (Fig. 1). In neonates, however, this clean separation between WM, GM and CSF does not occur naturally. At term-equivalent age, the average signal in cortical grey matter is nearly indistinguishable from that in the corpus callosum (CC), while most of the peripheral white matter decays much faster with increasing b-value. As shown in Fig. 1, the variability in mean signal curves between different WM structures is higher than the difference between WM and cortical GM. This makes the separation of WM and GM difficult, but allows the investigation of differences in different WM structures. Furthermore, the WM signal characteristics exhibit a strong age dependence.

**Fig. 2:**
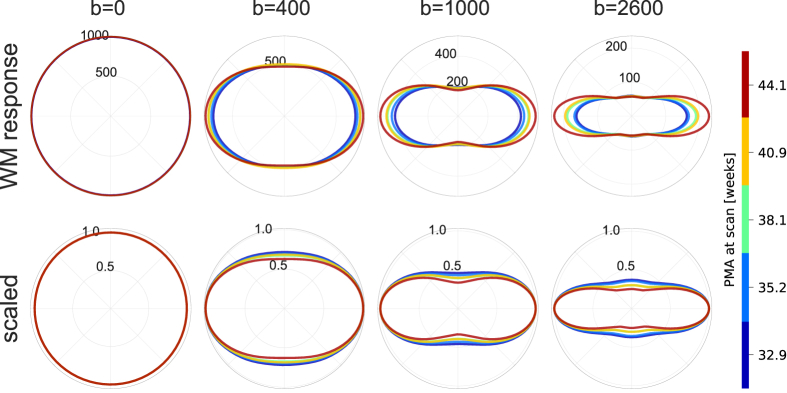
Longitudinal evolution of the WM response function of subjects scanned at 32.9 (n = 9), 35.2 (n = 11), 38.1 (n = 11), 40.9 (n = 11) and 44.1 weeks PMA (n = 11). Top: Shape and size change visualised as 2D projections through the fibre axis for each shell. Bottom: Each response function scaled independently at each b-value to unit radius to visualise the change in shape. b-values are in units of $s/mm^{2}$.

The WM response function estimated from each subject individually shows a clear age trend in the DC signal (mean signal or coefficient of the l=0 harmonic degree) and the higher degree harmonic coefficients between 32.9 and 44.1 weeks PMA (Fig. 2). With increasing age, the WM response function increases in sharpness and the signal decay across b-values reduces. Note that these weekly response functions exhibit distinct b-value dependencies and are not scaled versions of a single response function. This suggests that at least two components are required to model the WM signal in neonates accurately, and motivates the use of two anisotropic response functions as was performed in this study.

Given the approximately linear temporal evolution of the WM response functions as a function of age, we postulate that WM maturation can be modelled as a weighted sum of two response functions. We use the WM response function of the youngest and oldest age group of the cohort, $R_{\text{WM},y}$ and $R_{\text{WM},o}$ respectively, to test whether we can express the WM response function $R_{\text{WM},t}$ at any age *t* as a linear combination of those response functions by solving the least squares problem:

(1) $${\underset{\alpha ,\;\beta }{\text{arg}\;\text{min}}\left(\alpha *R_{\text{WM},y}+\beta *R_{\text{WM},o}-R_{\text{WM},t}\right)}^{2}$$

**Fig. 3:**
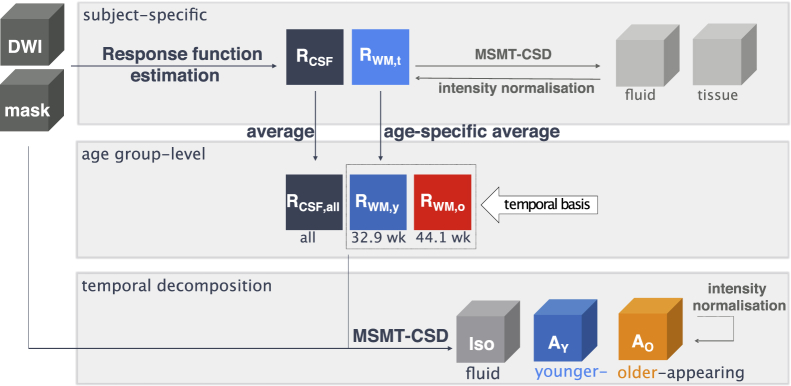
Overview of the estimation of the group-average response functions and the decomposition into time-resolved components. See section 2.4 for details.

Based on these observations and the results presented in 3.1, we chose to decompose the diffusion signal using one isotropic response function, derived from CSF voxels, and the two anisotropic response functions $R_{\text{WM},y}$ and $R_{\text{WM},o}$ (both with spherical harmonics degree 8) into the respective component images Iso, $A_{y}$, and $A_{o}$. See Fig. 3 for an overview of the response function estimation and image decomposition steps. This three tissue model serves as a basis to build a time-resolved group average template of white matter development, where the balance between the two WM components can be interpreted as an indication of the transition from immature to more mature tissue.

#### 2.4.1 Response function voxel selection

The CSF response function was estimated from voxels selected based on their average signal decay within a dilated full brain mask, using the method described in (Dhollander et al., 2016). The WM response functions were estimated from single-fibre voxels, identified using an iterative procedure (Tournier et al., 2013). This was performed with eroded brain masks to ensure only voxels within deep WM were selected. Briefly, the algorithm performs a single-shell CSD using a predefined initial response function; voxels where the main fibre orientation is most dominant are then selected, and an updated response computed by averaging the corresponding dMRI signal after realignment to a common fibre axis. This process is then repeated until convergence. For younger subjects, brain masks were eroded to exclude most of the cortical GM and single fibre voxel masks were edited manually to remove high FA voxels found in the remaining cortical voxels of younger subjects.

**Fig. 4:**
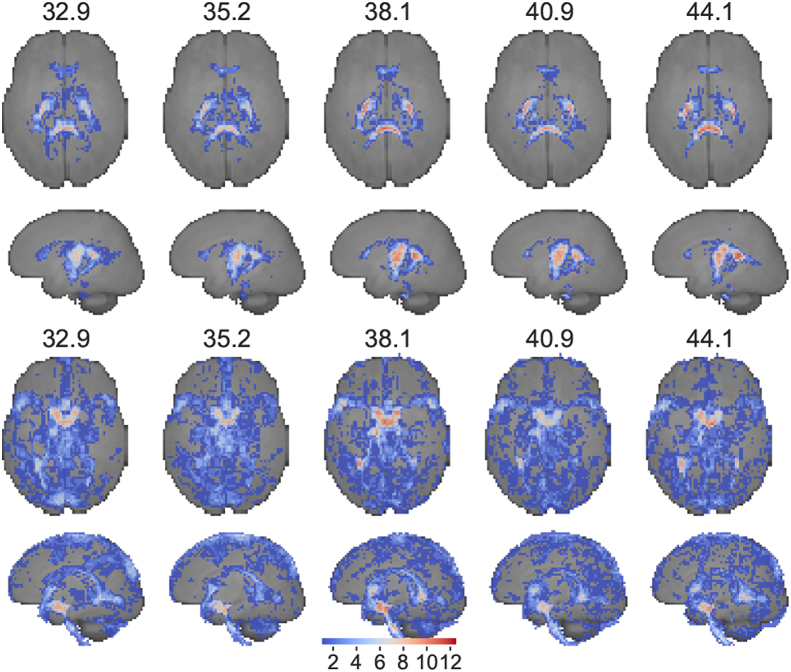
Maximum intensity projection of WM (top) and CSF (bottom) voxel selection masks. Colours represent the frequency with which a voxel has been selected in the respective weekly template. All images are nonlinearly aligned to a common space.

This resulted in consistent WM and CSF voxel selection maps across the age range, as shown in Fig. 4.

### 2.5 Bias field correction and intensity normalisation

As previously mentioned, the preprocessing of the data includes a bias field correction step (performed using ITK's *N4* algorithm), to minimise any potential influence on the estimated response functions. Any residual bias fields were subsequently corrected following MSMT-CSD by ensuring that the summed density of the three components has an average value of $\sqrt{1/\left(4\pi \right)}$; this was performed using the *mtnormalise* command available as part of *MRtrix3* (Raffelt et al., 2017b).

### 2.6 Multi-contrast ODF registration

A prerequisite for group-level or longitudinal analysis of HARDI data is unbiased and accurate spatial alignment. We use a symmetric non-linear diffeomorphic registration framework that takes the appropriate ODF reorientation into account (Raffelt et al., 2011) to align individual images to the respective group average image. The registration cost function metric is the squared $L_{2}$ norm of the spherical harmonics coefficients after reorientation between the image and the template, evaluated in the midway-space.

We extended the existing ODF registration framework (Raffelt et al., 2011) to allow multiple ODFs to be used simultaneously to drive the registration. This is done by using the weighted average of each contrast's contribution to the cost function to update the transformation in the gradient descent. In adults, using WM and GM compartments simultaneously (with equal weights) has been shown to yield higher registration accuracy and sharper features in the spatial and angular domain (Pietsch et al., 2017a). However, as previously mentioned, a decomposition into distinct GM and WM components is not effective for neonates, prompting us to decompose the tissue into immature and more mature anisotropic components. The problem for registration is that the boundaries between mature and immature tissue are age dependent; using all components would, therefore, bias the spatial alignment.

**Figure A.14:**
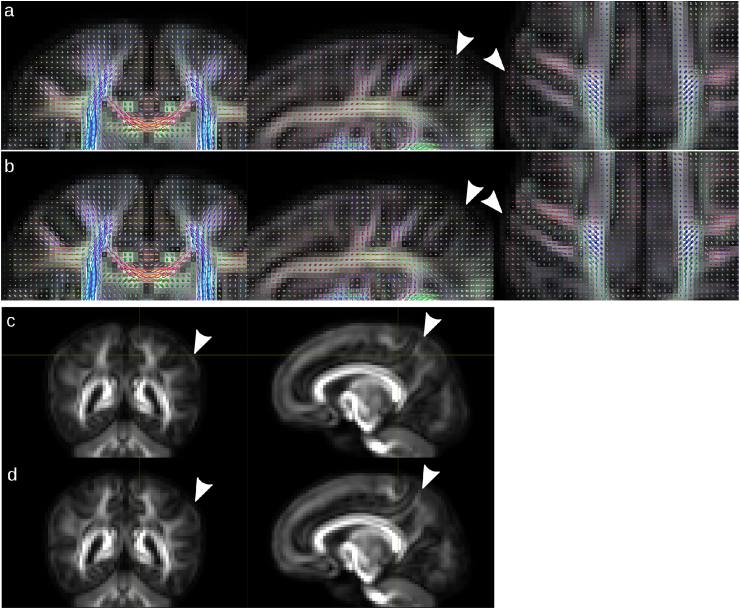
Comparison of templates of registration driven by WM only (a,c) with registration driven by the CSF and WM components simultaneously (b,d). The background image is the power in the second spherical harmonic degree. Arrowheads point to positions where the joint registration clearly yields sharper features. The image is an average of 27 subjects scanned at term equivalent age.

For this reason, we decided to use a simpler, two-component decomposition to drive the registration, obtained using responses consisting of each subject's native WM response, and the group average CSF response. Across age groups, the CSF component is consistently located primarily in the ventricles, whereas the single WM component covers the whole brain except for the ventricles and contains the orientational information necessary for the alignment of WM bundles. We find that using the CSF and native WM component for group alignment produces sharper templates (see figure A.14) compared to registration using the native WM component only.

We calculated the deformation fields for each subject by registering each subject's CSF component and single native WM component with equal weights to the respective two component version of the template, and subsequently applied those warps to the subject's three component decomposition to create the final three component atlas.

### 2.7 Group average template creation

We created unbiased weekly templates by iteratively averaging the respective registered images to the corresponding group average template for that week. The templates were created in 28 stages, with increasing degrees of freedom for the transformation and with increasing spatial and angular resolution. More specifically, these stages consisted of six rigid followed by six affine registration stages, each with decreasing voxel sizes from 3.3 mm to 1 mm and increasing angular resolution (highest harmonic degree $l_{max}=2$ for the first four, followed by four with $l_{max}=4$), followed by 16 nonlinear registration stages. The nonlinear registration increases spatial resolution in eight steps from 3.3 mm to 1 mm voxel size using $l_{max}=2$, followed by eight iterations with $l_{max}=4$ at full spatial resolution. For each iteration in each stage, the update and displacement fields are smoothed using a Gaussian kernel with a standard deviation of 2.0 and 1.0 times the size of the stage's voxel size, respectively. Warps are upsampled using linear interpolation if the resolution is increased between stages. Each image is registered to the group average formed from all other images, *excluding* the current image (leave-one-out) to ensure faster convergence.

Finally, we built a common template that aligns all images to a common space to visualise and analyse temporal variability of the three components. The creation of this joint atlas is identical to the creation of the separately aligned weekly templates. We use the resulting transformations to build weekly templates that are all aligned to the same space. Note that following the pre-processing, images are interpolated only once to transform them to their respective template space.

### 2.8 Obtaining quantitative density values

In MSMT-CSD, the mapping from HARDI signal to ODF amplitude is linear and the ODFs obtained are not inherently normalised, which necessitates the use of bespoke normalisation and bias field correction procedures to provide quantitative density values that can meaningfully be combined into a single analysis, such as this atlas. It also requires the use of a single set of responses that are appropriate over the entire age range, so that density estimates can be compared like for like.

First, we estimated CSF and WM response functions for each subject independently, following an initial coarse bias field correction. Those are used to deconvolve the signal into two components which are then subsequently corrected using a more fine-grained bias field correction using *mtnormalise* (*MRtrix3*). The two ODF images are jointly scaled so that they sum on average to $\sqrt{1/\left(4\pi \right)}$ within the subject's brain mask. For any further analysis, the subject's response function is also normalised using the inverse of the scale factor applied in the final bias correction. While not strictly necessary for the creation of the atlas, this procedure ensures that the estimated response functions are comparable across age-groups (Fig. 1).

Following the normalisation of the two subject-specific response functions, we average the normalised CSF response functions of all subjects and the normalised WM response functions of the youngest and oldest groups of the cohort and use those to deconvolve the initial bias field corrected dMRI images. We, therefore, use the same three response functions for all subjects. The final three component decomposition is bias-field corrected and normalised using *mtnormalise*. This ensures that ODF amplitudes are comparable across all subjects irrespective of their age.

### 2.9 Regions of interest selection

**Figure A.15:**
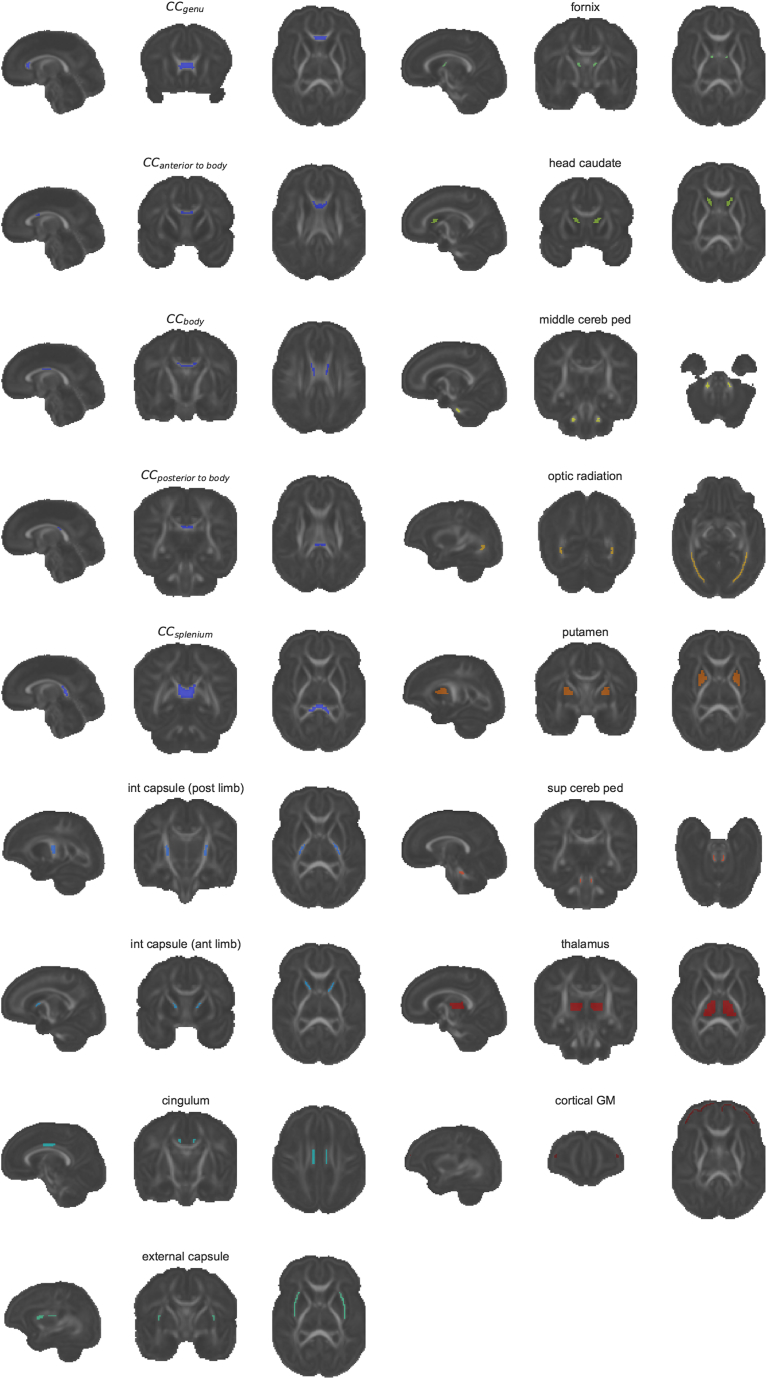
Slices showing all regions of interest, individually overlaid onto the age-average FA image. The focus of the projections coincides with the centre of mass of each region of interest.

We selected 17 regions of interest in white and grey matter structures. The areas included five regions in the CC from the genu to the splenium, along with further regions in the posterior and anterior limb of the internal capsule, the cingulum, external capsule, fornix, head of the caudate, middle cerebellar peduncles, optic radiation, putamen, superior cerebellar peduncles, thalamus and cortical GM (see figure A.15). In these regions of interest, the mean and standard deviation of the component densities were calculated for each weekly template to extract time-resolved developmental patterns.

Each ROI was defined by S.J.C. and M.P. on the jointly aligned group average template using the average FA and average component volume fraction maps and a consensus was reached. Furthermore, we consulted the diffusion tensor brain atlas (Oishi et al., 2005) for reference. Note that since the weekly templates are implicitly aligned with the average template, these regions are inherently applicable across all time points. To ensure temporal consistency across weekly templates, we refine each region of interest in the common average template so that they cover the core of the structure of interest in all weekly resolved FA and three tissue component maps.

## 3 Results

### 3.1 Multi-tissue model component selection

**Fig. 5:**
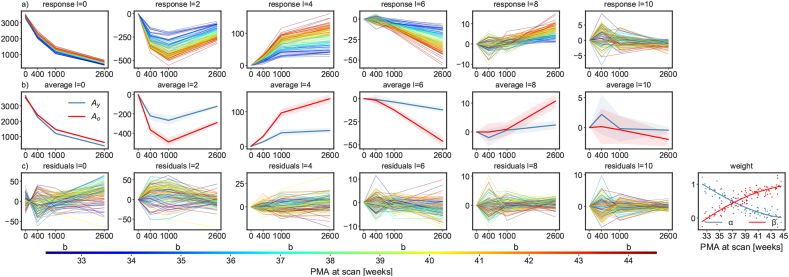
Evolution of the WM response in spherical harmonics coefficients in arbitrary units for harmonic degrees up to l=10. a) WM response function coefficients for each image in the cohort coloured by age at scan. b) WM response function average and 68% confidence interval of 9 neonates scanned at 32.9 weeks ($A_{y}$) and of 11 neonates scanned at 44.1 weeks PMA ($A_{o}$). The plots in row c) show the residuals (fit - data) and weights (*α* and *β*) of the linear model fit defined in equation (1). Weights are not constrained to be non-negative. The two curves for *α* and *β* are cubic polynomials fitted using a Huber kernel. b-values are in units of $s/mm^{2}$.

Fig. 5 shows that any response function in the cohort is well represented by a positive weighted sum of the two average response functions of the two age extremes of the cohort. The relative weight between the response functions transitions smoothly from the youngest to the oldest group, suggesting that MSMT-CSD performed using these responses can give meaningful separation of maturation patterns in WM, with the balance of density between the estimated weights for the two responses representing the level of WM maturation.

**Fig. 6:**
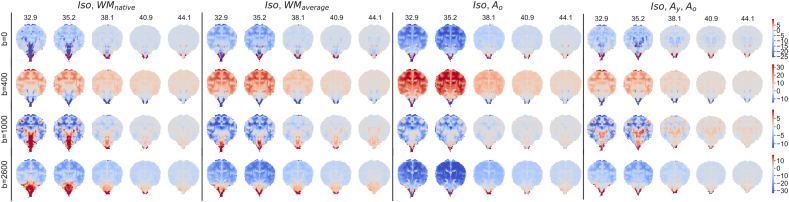
Residuals of the average signal in each shell for different response function combinations. Values are in percent relative to the average b = 0 signal. Iso refers to decomposition using the cohort average CSF response function, ${\text{WM}}_{native}$ to using the subject's WM response function, ${\text{WM}}_{average}$ to using the cohort average WM response function and $A_{y}$ and $A_{o}$ to decomposition using the average WM response function of the youngest and oldest cohort, respectively. b-values are in units of $s/mm^{2}$.

The appropriateness of the model was also investigated by looking at the residuals of each MSMT-CSD fit across the age range, as shown in Fig. 6. Using the three component decomposition yields lower residuals than any of the two-component models, which included the CSF response function (Iso component) and a single WM response function (including notably the case where each subject's native WM response function is used). However, there nevertheless remains anatomical structure in the residuals for the three component decomposition, indicating that the data may contain further information that is not captured by our model.

### 3.2 Group-level observations

**Fig. 7:**
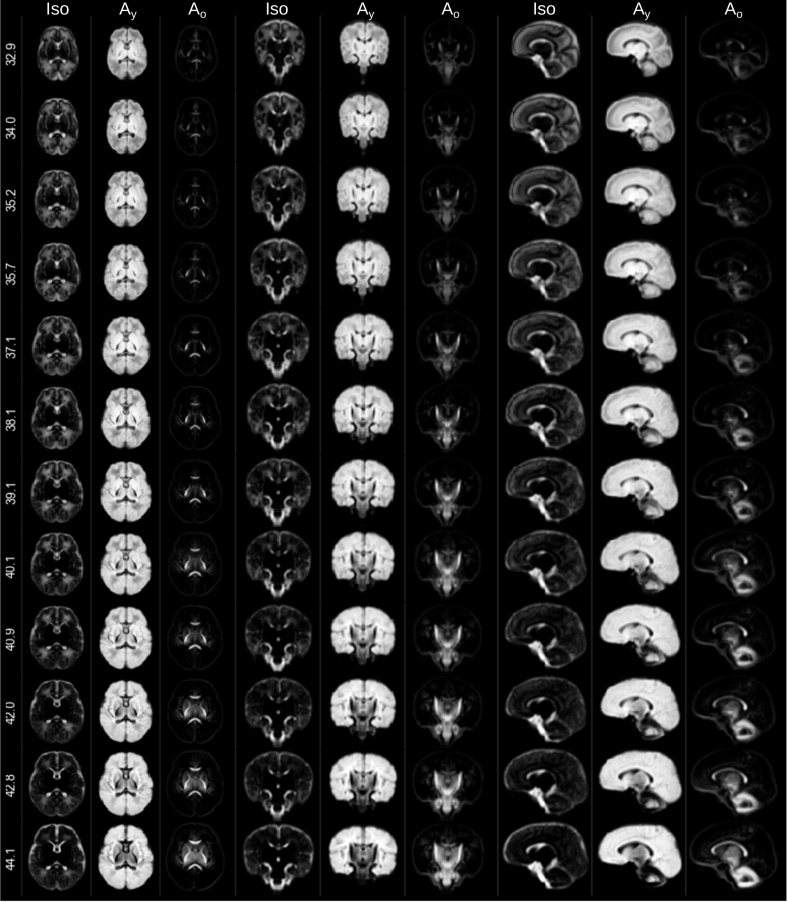
Display of changes in component volume fractions in weekly steps with image intensities scaled identically. Note that different anatomical orientations are scaled differently in size.

**Fig. 8:**
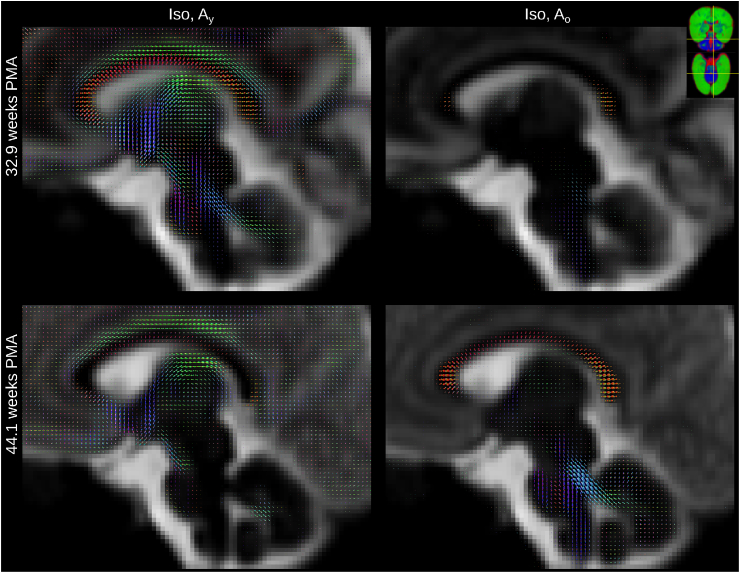
Sagittal sections showing the isotropic component (background) and the two anisotropic components through the brainstem at 32.9 (top row) and at 44.1 (bottom row) weeks PMA. All images are part of the jointly aligned atlas.

**Fig. 9:**
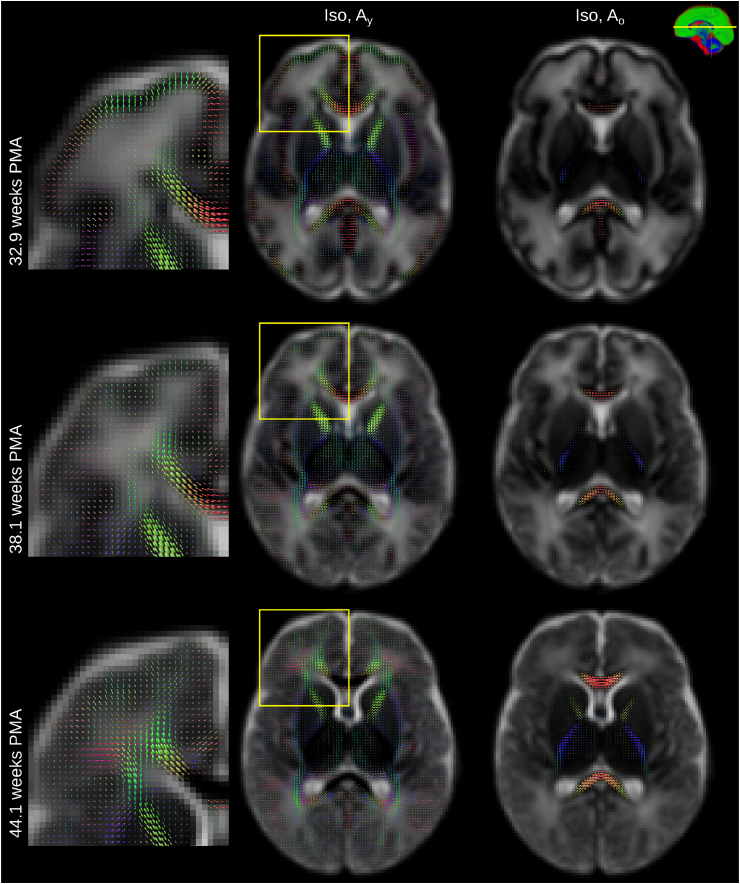
Axial sections showing the isotropic component (background) and the two anisotropic components through the CC and periventricular crossroads at 32.9 (top row) and at 44.1 (bottom row) weeks PMA. Magnified cropped images show high anisotropy in the cortical GM and high Iso component volume fraction in the periventricular crossroads observed in the young subjects. All images are part of the jointly aligned atlas.

The component volume fraction maps in Fig. 7 show the decreasing Iso content and the increase of the mature tissue component in brain parenchyma over time. This matches the expected decrease in overall brain tissue water content during development (Dobbing and Sands, 1973). In our decomposition, early maturing WM such as the cerebellum, cerebellar peduncle or CST exhibit high $A_{y}$ and $A_{o}$ and low Iso density at all ages (see Fig. 8). In contrast, parts of the periventricular deep white matter show relatively high free water content (see Fig. 9).

In general, the transition from young to mature-appearing WM occurs from central to peripheral, caudal to cranial and posterior to anterior. The slices shown in Fig. 7 display this pattern most prominently in the periventricular crossroads, the CST and the CC. The sagittal images exhibit a pattern of transition from young to more mature appearing WM in the cerebellum and the CC, consistent with the expected behaviour (Branson, 2013).

Fig. 8 shows the spatially localised $A_{o}$ component in the youngest cohort. At 32.9 weeks PMA, it is confined nearly exclusively to the genu and splenium of the CC and WM in the CST, the spine, parts of the midbrain and the cerebellum. The transition from $A_{y}$ to $A_{o}$ is similar for the genu and splenium of the CC but the body of the CC has a comparatively high density of $A_{y}$ fibres even at 44.1 weeks PMA.

In the cortex of younger subjects, we observe clear radial organisation (Fig. 9) which reduces with age, consistent with the known process of cortical formation. Anisotropy in this area has been shown to drop as dendritic arborisation proceeds (Miller et al., 2011; McKinstry et al., 2002).

## 4 Discussion

The diffusion tensor model has been used extensively to study brain maturation (Yoshida et al., 2013; Miller et al., 2003; Hüppi and Dubois, 2006) but current higher order models can capture temporal patterns in neonatal HARDI data with higher fidelity (Genc et al., 2017) and potentially give access to richer microstructural properties. Current approaches to microstructural modelling generally fit the data well but suffer from a lack of specificity and they rely on implicit or explicit assumptions that might not be met (Novikov et al., 2018) – especially in neonatal cohorts. We propose a framework facilitating the group-level analysis of diffusion MRI data over the neonatal period, based on recent advanced models of microstructure that provide a data-driven decomposition of the signal. We sample *response functions* in CSF and in WM in the extremes of the age-range to model temporal changes in signal characteristics as changing relative volume fractions.

Group-level and longitudinal analysis of these decomposition patterns requires spatio-temporal alignment. We adapted an existing registration approach, which allows the groupwise alignment of ODFs, to neonatal data. Registration is driven by two signal components, taking advantage of the information in both channels without biasing the alignment of the 3-component model due to spatial variability in maturation patterns. This template creation strategy does not explicitly model temporal consistency but could be extended to incorporate temporal shape and intensity constraints similar to atlases discussed in section 1.1. The major discriminating feature of our approach is the explicit time-associated property of the contrast itself, which can be embedded into longitudinal models in future work.

Our approach allows a group-level and longitudinal analysis of white matter regions or tracts of interest in the neonatal period using components that are derived from the data and not modelled after biological tissue properties. While it does not offer the level of biological specificity that more explicit microstructure models might provide, it relies on very few assumptions, which we believe is a strong advantage given that the microstructure changes occurring over this period are complex and likely to differ substantially from a priori expectations.

### 4.1 Longitudinal component volume fraction changes in selected regions

Histology studies have reported spatially varying onset and progression of myelination (Brody et al., 1987; Gilles et al., 1983). Myelination progresses in a nonlinear and location-specific manner starting at the end of the fourth foetal month lasting until adulthood in the CC (Kinney et al., 1988). However, myelinogenesis is preceded by complex changes in cellular constituents and their organisation in the premyelinating stages (Back et al., 2002; Wimberger et al., 1995).

We investigate regional differences of WM maturation patterns similarly to earlier studies which used diffusion-weighted imaging (Bui et al., 2006), diffusion tensor imaging (Zanin et al., 2011) or HARDI (Kunz et al., 2014). Our work differs from this early work in that we use tissue-specific responses instead of biophysical model quantities. Furthermore, in contrast to tensor-based work, our approach can resolve multiple fibre populations within a single voxel. In fact, in some crossing fibre regions, the different bundles are ascribed to different anisotropic responses, potentially reflecting different stages of maturation for the different bundles (Fig. 11).

**Fig. 10:**
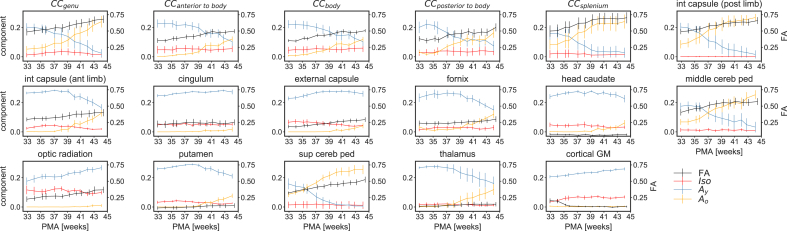
Longitudinal changes of component volume fractions and FA in selected WM and GM regions of interest. Error bars represent one standard deviation across voxels in the respective region and are calculated in the average space.

**Fig. 11:**
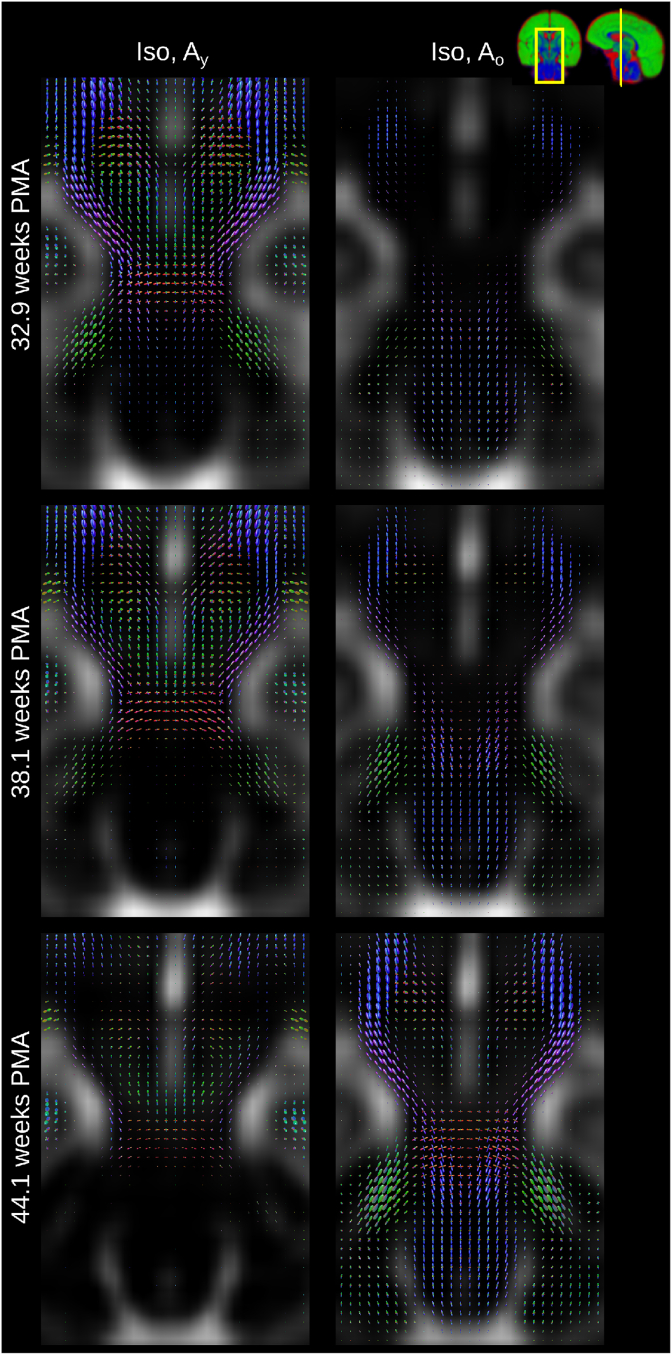
Coronal sections showing the isotropic component (background) and the two anisotropic components through the CST and brainstem at 32.9 (top), 38.1 (middle) and at 44.1 (bottom) weeks PMA. Note the transition from $A_{y}$ to $A_{o}$ is different for different fibre populations within the same voxel. All images are part of the jointly aligned atlas.

The middle and superior cerebellar peduncles (see Fig. 10) exhibit a relatively high fraction of $A_{o}$ at 33 weeks PMA, which increases almost linearly until term (superior) and 44 weeks (middle). Of interest, the superior cerebellar peduncle has a higher fraction of $A_{o}$ from 33 weeks which is consistent with the earlier maturation of the superior cerebellar peduncle compared to the middle cerebellar peduncle (Gilles et al., 1983).

We observe that in the posterior limb of the internal capsule, the relative fraction of the $A_{o}$ component increases rapidly from 33 weeks until 40 weeks after which it slowly increases until 44 weeks. This is in contrast to the anterior limb of the internal capsule which starts to transition from $A_{y}$ to $A_{o}$ only after 39 weeks. This is in agreement with reported temporal maturation time courses for these two adjacent structures, as myelin is present in histological sections of the posterior limb of the internal capsule starting at 34 weeks, and myelinates rapidly until after term (Gilles et al., 1983), whereas the anterior limb of the interior capsule shows no evidence of myelination until after term (Gilles et al., 1983).

In comparison, the external capsule, the fornix, the cingulum and the optic radiations do not myelinate before term (Gilles et al., 1983; Yakovlev and Lecours, 1967). We also observe a later onset of increasing $A_{o}$ volume fractions in those structures.

In the CC, the splenium appears to mature before the genu and the body exhibits a more protracted maturational pattern. The splenium has been observed to mature before the genu on T_1_-and T_2_-weighted imaging (Barkovich et al., 1988).

The pattern of maturation in deep GM is distinct from that of early maturing WM. The head of the caudate nucleus, the putamen and the thalamus contain very little $A_{o}$ signal until 37 weeks PMA. The mature component rises more steeply in the thalamus which matches observed myelogenesis in the last trimester (Yakovlev and Lecours, 1967).

### 4.2 Limitation of the three tissue model

We focus on modelling the spatial variability of longitudinal changes in WM. The components of our atlas were chosen to be interpretable in terms of brain maturation. However, WM maturation is undeniably a complex biological process, giving rise to dMRI signals that might not necessarily be fully characterised using three components alone. Nonetheless, our approach is likely to provide a good first-order approximation to the dominant effects observed in the data over this age range.

It is important to note that the anisotropic WM responses used in this study correspond to the extremes of the age range under consideration, and are therefore inherently dependent on these ages.

Also, the use of CSF (Iso) and two WM response functions is not necessarily applicable to the rest of the brain parenchyma. This is apparent in the MSMT-CSD residual maps of the younger cohorts (Fig. 6). Yet, this work is the first study using a data-driven approach to describe WM maturation during the perinatal period in a fibre-resolved manner. Improving on the response functions selection to model the full brain is scope for future work.

### 4.3 Multiple fibre specific maturation patterns in a voxel

Differentiating between distinct fibre populations within a single voxel based on differences in microstructural features is an ongoing challenge in dMRI. Microstructure-informed tractography methods have been proposed to disentangle multiple fibre populations (Daducci et al., 2015; Sherbondy et al., 2010; De Santis et al., 2016).

**Fig. 12:**
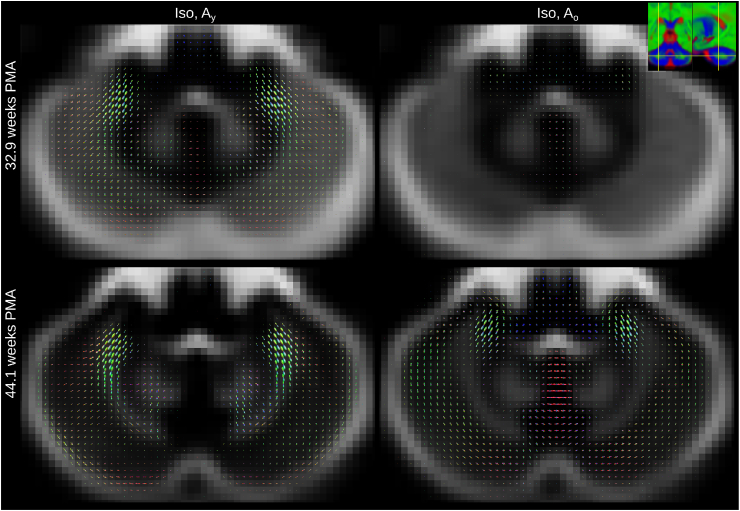
Axial sections showing the isotropic component (background) and the two anisotropic components through the cerebellar dentate nucleus at 32.9 (top row) and at 44.1 (bottom row) weeks PMA. All images are part of the jointly aligned atlas.

MSMT-CSD allows resolving multiple tissue types in the same voxel. Using two anisotropic response functions, we can directly resolve fibre populations from different components in the same voxel if the fibre populations are separable using the chosen response functions. We observe this for instance in the cerebellum. Our three-component model separates fibres in cerebellar GM that follow a radial trajectory from tangential fibres within the same voxel (Fig. 12). This matches with observations of radial and tangential pathways (Takahashi et al., 2014) that mature at different rates in the cerebellum.

Furthermore, Fig. 11 shows a section through the CST and the midbrain which illustrates that the “maturation” trajectory for fibres going in craniocaudal direction is distinct from that of pontocerebellar fibers in the same voxel. Resolving multiple maturation patterns in a voxel opens new possibilities for longitudinal investigations in a fibre-specific manner using frameworks such as fixel-based analysis (Raffelt et al., 2017a). Note that this ability to resolve different fibre populations based on their distinct microstructural signature is possible due to the large differences that brain development introduces in their dMRI signature; differences of such magnitude are unlikely to be observed in adult data.

## 5 Conclusions

We propose a method to create a time- and orientation-resolved multi-tissue group average template of the neonatal brain using three components derived from CSF, WM at 32.9 and WM at 44.1 weeks postmenstrual age. We demonstrate this approach to build an atlas of brain development using data from the dHCP cohort, from which we can observe regionally-varying temporal patterns in the transition between the young (32.9 weeks PMA) and more mature (44.1 weeks PMA) appearing anisotropic components. Furthermore, we were able to distinguish fibre populations within the same voxel with distinct time courses. This framework provides a basis for longitudinal investigations into healthy and pathological brain maturation.

## Footnotes

1 http://www.developingconnectome.org/.
